# Nasal delivery of a CRMP2-derived CBD3 adenovirus improves cognitive function and pathology in APP/PS1 transgenic mice

**DOI:** 10.1186/s13041-020-00596-3

**Published:** 2020-04-09

**Authors:** Baochang Qi, Yu Yang, Yingying Cheng, Di Sun, Xu Wang, Rajesh Khanna, Weina Ju

**Affiliations:** 1 Department of Orthopedic Traumatology, The First Hospital of Jilin University, Changchun, 130021 Jilin Province China; 2 Department of Neurology and neuroscience center, The First Hospital of Jilin University, No.1 Xinmin Street, Chaoyang District, Changchun, 130021 Jilin Province China; 3 Department of Colorectal and Anal Surgery, The First Hospital of Jilin University, Changchun, 130021 Jilin Province China; 4 Department of Pharmacology, College of Medicine, The University of Arizona, Tucson, AZ 85718 USA; 5 Center for Innovation in Brain Sciences, University of Arizona, Tucson, AZ 85721 USA

**Keywords:** Alzheimer’s disease, Calcium channel-binding domain 3, Apoptosis, APP/PS1 mice, Amyloid beta

## Abstract

Calcium dysregulation is a key pathological event in Alzheimer’s disease (AD). In studying approaches to mitigate this calcium overload, we identified the collapsin response mediator protein 2 (CRMP2), an axonal guidance protein that participates in synapse dynamics by interacting with and regulating activity of N-methyl-D-aspartate receptors (NMDARs). We further identified a 15 amino acid peptide from CRMP2 (designated CBD3, for calcium-binding domain 3), that reduced NMDAR-mediated Ca^2+^ influx in cultured neurons and post-synaptic NMDAR-mediated currents in cortical slices. Whether targeting CRMP2 could be therapeutically beneficial in AD is unknown. Here, using CBD3, we tested the utility of this approach. Employing the APP/PS1 mouse model of AD which demonstrates robust pathophysiology including Aβ1–42 deposition, altered tau levels, and diminished cognitive functions, we asked if overexpression of CBD3 could rescue these events. CBD3 was engineered into an adeno-associated vector and nasally delivered into APP/PS1 mice and then biochemical (immunohistochemistry, immunoblotting), cellular (TUNEL apoptosis assays), and behavioral (Morris water maze test) assessments were performed. APP/PS1 mice administered adeno-associated virus (AAV, serotype 2) harboring CBD3 demonstrated: (i) reduced levels of Aβ1–42 and phosphorylated-tau (a marker of AD progression), (ii) reduced apoptosis in the hippocampus, and (iii) reduced cognitive decline compared with APP/PS1 mice or APP/PS1 administered a control virus. These results provide an instructive example of utilizing a peptide-based approach to unravel protein-protein interactions that are necessary for AD pathology and demonstrate the therapeutic potential of CRMP2 as a novel protein player in AD.

## Introduction

Alzheimer’s disease (AD) has been regarded as an age-dependent neurodegenerative disease, and one of the most common forms of dementia, clinically manifesting as progressive cognitive and behavioral impairment [46]. The prevalence of AD generally doubles every 5 years in adults over the age of 65 [31], and it has been estimated that about 152 million people will be living with AD by 2050 [44]. Multiple factors, such as vascular risk factors, lifestyle, and genetic susceptibility have been reported to be involved in AD pathogenesis. The neurofibrillary tangles (NFTs) composed of hyperphosphorylated tau protein and senile plaques consisting of amyloid beta (Aβ) peptide are the neuropathological hallmarks of AD [25, 45, 51]. AD leads to a progressive decline in the quality of life of patients, including a barrier in communication skills, increased incidence of wandering, and an inability to recognize familiar faces [33]. To improve the understanding of AD pathogenesis and explore potential curative treatments for it, the amyloid precursor protein / presenilin 1 (APP/PS1) mouse model was constructed to simulate the behavioral and pathological changes observed in AD patients [35, 57].

Among the earliest neuropathological changes in AD is the degeneration of neurons likely arising from the deposition of β-amyloid plaques as well as neurofibrillary tangles in the brain. Neuritic degeneration, including neuronal dysfunction and loss of functional synapses, can lead to cognitive and memory dysfunction, predominantly in elderly people. Thus, targeting proteins that cause disruption of the neuronal circuitry may be a therapeutically useful means to ameliorate the harmful consequences of neurotoxicity in AD. Previous studies identified high levels of phosphorylated collapsin response mediator protein 2 (CRMP2) [23], an axonal specification protein that also regulates microtubule assembly, in neurofibrillary tangles and abnormal neurites from human AD brains, suggesting that accumulation of CRMP2 may be an early event in AD [20]. Increased CRMP2 levels have been reported in human AD brains [14, 19] in association with neurofibrillary tangles [62]. Importantly, a recent study showed that CRMP2 is an important signaling molecule for prevention of β-amyloid induced memory loss [37]. Therefore, these studies suggested that CRMP2 may be a novel target for neuroprotection in AD.

CRMP2 has been demonstrated to modulate Ca^2+^ influx through activation of presynaptic voltage-gated calcium channels (VGCCs): CRMP2 augmented capacity of hippocampal neurons to release neurotransmitters through enhancement of both Ca^2+^ channel trafficking and Ca^2+^ currents [10, 18, 58]. Regulation of postsynaptic receptors by CRMP2 has also been reported via its interaction and modulation of N-methyl-D-aspartate receptors (NMDARs) [2, 8], with a preferential interaction for GluN2B-containing NMDARs. Notably, Ca^2+^ dysregulation, presumably via both VGCCs and NMDARs, has been hypothesized as a possible mechanism for the observed AD-related neurodegeneration [52]. Excessive activation of NMDARs is linked to a drastic elevation in cytosolic Ca^2+^ concentration, which activates Ca^2+^-dependent degradation enzymes such as phospholipases and proteases [26, 53]. The Ca^2+^-activated protease calpain has been shown to cleave NR2B after excitotoxic glutamatergic stimulation [24, 48]. Most relevant to our work, it was reported that a cleaved form of CRMP2 caused a reduction in levels of surface NR2B [8]. In other words, calpain-cleaved form of CRMP2 reduces the amount of surface expressed NR2B which accounts for the neuroprotective effects during Aβ-induced excitotoxic insults. Aβ_25–35_ oligomers can directly trigger NMDA receptor function, elevating intracellular Ca^2+^ and causing neuronal toxicity or death [55]. Importantly, a recent study showed that CRMP2 is required in an early stage of memory consolidation [37], thereby providing a crucial link between CRMP2 and AD.

In this context, we identified a peptide designated the calcium channel-binding domain 3 (CBD3) peptide from CRMP2. This peptide, made cell permeant by addition of a charged sequence from the Transactivator of transcription (TAT) protein of HIV, inhibited the NMDAR-CRMP2 interaction, reduced NMDAR-mediated Ca^2+^ influx in cultured neurons and post-synaptic NMDAR-mediated currents in cortical slices [9, 11, 13, 34]. The peptide’s actions occurred via activity-dependent down-regulation of GluN2B-containing NMDAR surface expression in dendritic spines, providing neuroprotection against excitotoxic neuronal loss in multiple rodent models [9, 11, 13, 64]. In addition to its neuroprotective actions, the TAT-CBD3 peptide was demonstrated to be antinociceptive across a variety of acute and chronic pain models [22, 29, 41, 47]. Moreover, recombinant adeno-associated viral (AAV)-mediated expression of the wildtype CBD3 or a derivatized version of the peptide targeted to the peripheral sensory nervous system prevented the development of pain hypersensitivity after peripheral nerve injury [21, 63], supporting the utility of long-term treatment of pathophysiological conditions using this peptide. Whether CBD3 could be used in a similar manner in altering the progression or hindering the development of AD remains unknown. The present study, therefore, aimed to explore the potential effect of CBD3 on novel object recognition and spatial memory, as well as on pathology development in APP/PS1 mice.

## Materials and methods

### Construction of the Adeno-associated virus (AAV) vectors

To construct the AAV (serotype 2) vectors coding for CBD3 or control, two gene segments NT4-TAT-CBD3 and NT4-TAT, respectively, were synthesized with the following primers: NT4-TAT-CBD3: ATGCTCCCTCTCCCCTCATGCTCCCTCCCCATCCTCCTCCTTTTCCTCCTCCCCAGTGTGCCAATTGAGTCCCAACCCCCACCCTCAACATTGCCCCCTTTTCTGGCCCCTGAGTGGGACCTTCTCTCCCCCCGAGTAGTCCTGTCTAGGGGTGCCCCTGCTGGGCCCCCTCTGCTCTTCCTGCTGGAGGCTGGGGCCTTTCGGGAGTCAGCAGGTGCCCCGGCCAACCGCAGCCGGCGTTATGGCAGGAAGAAGCGGAGACAGCGACGAAGAGCTCGTTCTCGCTTAGCCGAATTGCGAGGTGTTCCTCGGGGCCTT;

NT4-TAT (control): ATGCTCCCTCTCCCCTCATGCTCCCTCCCCATCCTCCTCCTTTTCCTCCTCCCCAGTGTGCCAATTGAGTCCCAACCCCCACCCTCAACATTGCCCCCTTTTCTGGCCCCTGAGTGGGACCTTCTCTCCCCCCGAGTAGTCCTGTCTAGGGGTGCCCCTGCTGGGCCCCCTCTGCTCTTCCTGCTGGAGGCTGGGGCCTTTCGGGAGTCAGCAGGTGCCCCGGCCAACCGCAGCCGGCGTTATGGCAGGAAGAAGCGGAGACAGCGACGAAGA. The PCR-amplified gene segments were then inserted into the empty pAAV-CAG-MCS-EGFP-3FLAG AOV002 vector (Supplementary Figure 1), which was double-digested by *EcoRI* and *BamHI*. The Flag epitope was used for immunoblotting to verify efficiency of virus transduction. The NT4 is a signal peptide that has been shown to enhance expression of proteins in eukaryotic cells [50]. The 6His tag was added for protein purification but not used in this work.

#### Construction and packaging of AAV2 vectors

The AAV used in our present study are based on serotype 2. The AAV Helper-Free System (Stratagene) was used for viral vector preparation. The AAV vector harboring NT4-TAT-CBD3 or NT4-TAT sequences were transferred into recipient bacterium (Top10 strain). The successfully transferred bacterial colony was selected by enzyme identification and was cultured to generate the viral. Three plasmids (pAAV/Ad, pAAV/Ad cofactor, pSSCMV-BDNF-HA2TAT) were transfected into HEK293 cells to generate virus AAV/ NT4-TAT-CBD3 or AAV/ NT4-TAT. The AdEasy Viral Titer Kit, a simple enzyme-linked immunoassay, was used for the determination of adenoviral titers (Agilent, Santa Clara, CA, USA).

### Cell culture and transfection

The HEK293TN Pseudoviral Lenti Particle Producer Cell Line cell (293TN) was purchased from the Institute of Biochemistry and Cell Biology (Chinese Academy of Sciences, Shanghai, China). The cells were maintained in RPMI-1640 medium (Gibco, CA, USA), supplemented with 10% fetal bovine serum (Gibco, CA, USA) and 1% penicillin-streptomycin at 37 °C in 5% CO2. The plasmid vectors were transfected into 293TN cells using Lipofectamine 2000 (Invitrogen, Carlsbad, CA, USA). At 48 h after transfection, the expression of the target gene was detected in the lysed cells.

### Animals and virus administration

All transgenic APP/PS1 mice and wild-type male mice (C57BL/C, 3–4 months old) were purchased from the Institute of Laboratory Animal Science, China Medical University, and bred in standard housing conditions (12 h alternating light and dark) with free access to distilled water and food. The feeding environment and bedding materials were sterilized every day. The mice were randomly divided into the following four groups (*n* = 13 in each group): (1) wild-type mice (WT); (2) APP/PS1 transgenic mice (APP/PS1); (3) APP/PS1 transgenic mice treated with empty vector (APP/PS1 + control); and (4) APP/PS1 transgenic mice treated with CBD3 (APP/PS1 + CBD3). A limitation of this design was the omission of two groups: wildtype mice treated with empty vector and wildtype mice treated with CBD3 overexpression. This study was approved by the Ethical Committee of the First Hospital of Jilin University.

The virus suspension (either control vector or vector containing CBD3) was administered to the APP/PS1 mice by nasal drip at 50 μ L per mouse. The virus titer was 1 × 10^13^/mL, and it was administered nasally once (virus per mouse was 5 × 10^11^/mL) and then again 20 days later. Mice were used 9 days after the double nasal infusion paradigm. Previous studies have shown intranasal administration to be a non-invasive and efficient means of delivering therapeutics to the brain (including the hippocampus) to treat neurodegenerative diseases including AD [3, 16, 36]. Increases in the hippocampi in mice intranasally administered the AAV-administered payload, demonstrated by immunoblotting, have been reported previously [36]; the increase was detected at 10 days post intranasal administration while our studies were conducted at 9 days post intranasal administration . After behavioral testing, the hippocampal tissues of some (*n* = 3–4) of the sacrificed mice were immediately collected for experimentation. The mice were anesthetized with pentobarbital sodium salt (40 mg/kg), then decapitated and the hippocampal tissues of sacrificed mice were immediately collected for experimentation.

### Morris water maze (MWM) test

The MWM test was conducted in the apparatus after virus administration on day 30; 9 days after the second nasal administration of the virus. The apparatus consisted of a circular polypropylene pool (120 cm in diameter and 40 cm in height) filled with white-opaque water (25 ± 1 °C). Briefly, the spatial learning task included 4 consecutive days of testing, the time required to find the hidden platform was defined as escape latency. In order to investigate the spatial memory, a single probe trial was performed 24 h after the final test on day 4. After the submerged platform was removed, the mice were placed into the pool in the quadrant opposite to the target quadrant. The video recording time was set as 60 s. Moreover, the time of the mice spent in the target quadrant, as well as the number of mice crossings through this quadrant were recorded and analyzed by SMART 3.0 software.

### Immunohistochemical staining and quantification

After baking at 62 °C for 1 h, the sections were dewaxed with xylene, dehydrated with an ethanol gradient, and then incubated with tris-EDTA (pH 9.0) for antigen retrieval. Subsequently, the primary antibodies, including p-tau (1:500; Abcam; ab75679), t-tau (1:500; Abcam; ab47579), and Aβ-42 (1:500; Abcam; ab201060) were applied to the sections at 4 °C overnight. After being washed with PBS, the sections were reacted with horseradish enzyme-labeled secondary antibodies (goat anti-mouse IgG (H + L); 1:1000; Jackson; 715–035-151) for 30 min at 37 °C. The positive signal was visualized with DAB. The sections were stained by hematoxylin (Zhuhai Beso Biotechnology Co. LTD; BA-4097) and imaged with an Optika inverted fluorescence microscope. Aβ1–42 plaque burden was calculated by examining hippocampal regions with positive DAB signals that appeared to be in plaque-like. At least three sections per condition from 3 separate animals each), at least 30 μm apart, and then the mean density of positive DAB signal appearing in clusters (brown was taken to determine plaque burden) was quantified per slice. The sections were chosen randomly; every third section (~ 30 μm apart) was selected from the initially random chosen slice. This is now indicated in the text. The data are presented as mean ± SD plaques per slice.

### Western blotting

Total proteins extraction from hippocampal tissues sections was performed using RIPA lysis buffer (Beyotime; P0013B), and then separated with SDS-PAGE gel and transferred onto the PVDF membrane. The membrane was blocked in 5% (0.75 g milk powder + 15 mL PBS) milk for 1–2 h and then incubated with primary antibodies as follows: phosphorylated tau (p-tau; 46 kDa; 1:500; Abcam, Cambridge, MA, USA); total tat (t-tau; 79 kDa; 1:200; Abcam, Cambridge, MA, USA); Aβ1–42 (40 kDa; 1:500; Abcam, Cambridge, MA, USA); ADAM10 (84 kDa; 1:1000; Abcam, Cambridge, MA, USA); and GAPDH (36 Da; 1:20000; Proteintech: 10494–1-AP) at 4 °C overnight. After washing in TBST for 3 times, the corresponding secondary antibodies (goat anti-mouse IgG (H + L); 1:1000; Jackson; 715–035-151) was applied to the membrane for 2 h at 37 °C. The band was visualized by the ECL (Share-Bio; SB-WB012). Western blots were quantified using Un-Scan-It gel version 6.1 (Silk Scientific Inc., Orem, UT).

### Terminal-deoxynucleotidyl-transferase mediated nick end labeling (TUNEL) apoptosis

The cell apoptosis assay was performed with TUNEL method. Briefly, after dewaxing treatment, hippocampal sections were placed in the TUNEL mixture (1 mL TUNEL buffer; 10 μ L b-11-DUTP; 10 μ L TDT) for 1 h at 37 °C. Slides were developed with 0.04% DAB and 0.03% H_2_O_2_ for 10 min, and then counterstained with 10% hematoxylin. The apoptotic cells were visualized and analyzed with Image-Pro Plus 6.0. A total of 3 random fields per hippocampal section from 3 individual mice from each treatment were analyzed. The data were presented as follows: the number of TUNEL-positive cells/total cell number× 100 (%).

### Statistical analysis

All data points are shown as mean ± S.D. For all Western blots n is presented as the number of separate experiments. Unless otherwise stated, statistical differences between control and experimental conditions were determined by using Kruskal-Wallis non-parametric test followed by Dunnet’s post hoc test or a Mann & Whitney non-parametric test when comparing only two conditions within R Software (R-project). *p* values < 0.05 were judged to be statistically significant.

## Results

### AAV vector construction and validation of the AD model

**Fig. 1:**
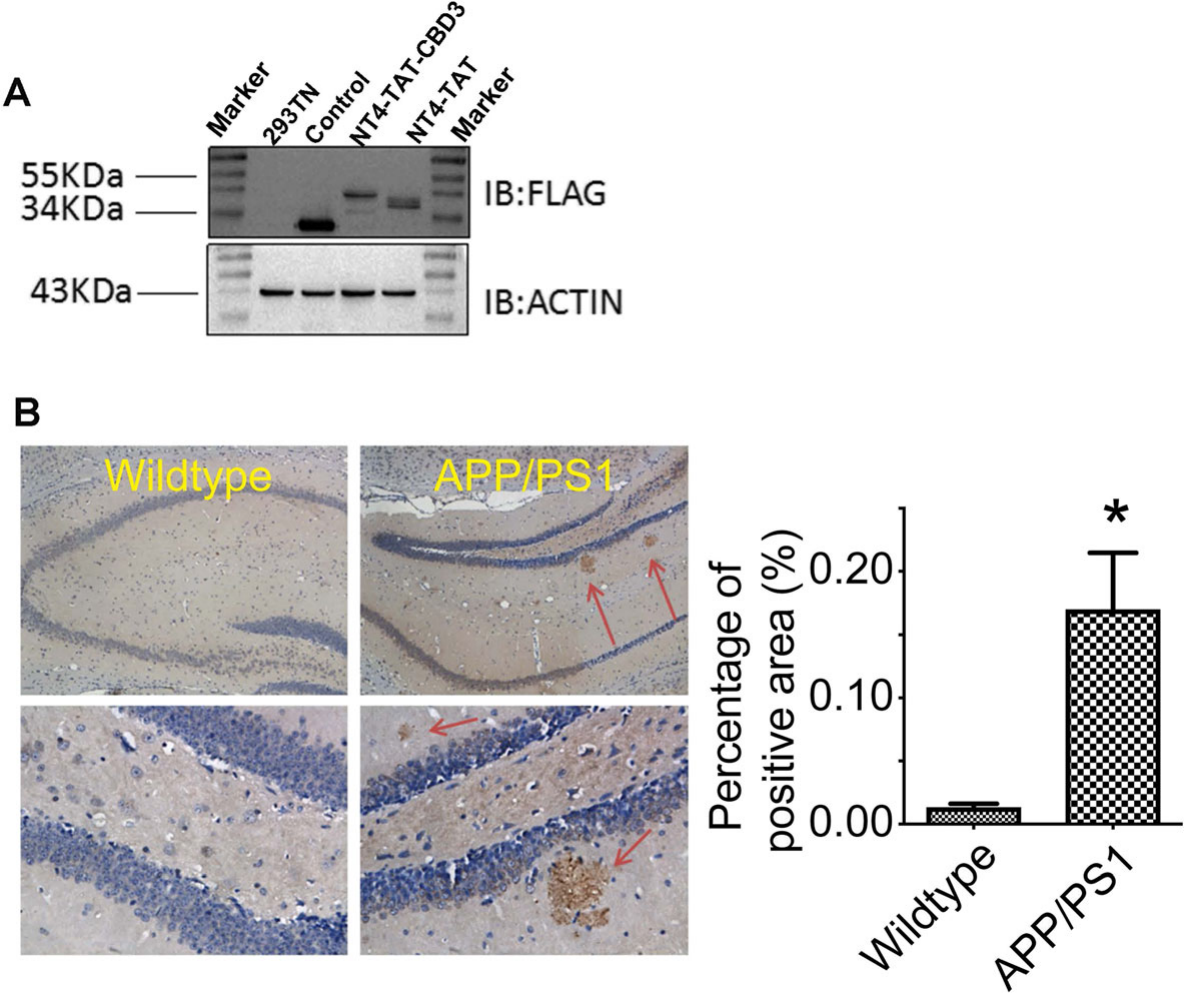
AAV vector construction and validation of AD pathology in APP/PS1 mice. **a** Lysates from HEK293TN cells transfected with the indicated constructs were immunoblotted with anti-Flag (*top*) or actin (*bottom*) antibodies. Representative blots are shown (*n* = 3). Expression of a NT4-TAT-CBD3 protein was noted running at a higher molecular weight than the NT4-TAT protein devoid of the CBD3 fragment. **b** In contrast to wildtype mice, APP/PS1 mice (male 3–4 months old) exhibit typical amyloid-beta aggregation as revealed by staining with an antibody against Aβ-42. Higher magnification images of the micrographs of the CA1–2 transitional field of the hippocampus. Representative of *n* = 3 for age-matched control and n = 3 for the APP/PS1 mice. *P < 0.05, Mann & Whitney non-parametric test

As shown in Fig. 1a, immunoblotting lysates from HEK293 cells transduced with the CBD3 or control viruses revealed the presence of a higher molecular weight protein in cells from AAV NT4-TAT-CBD3 infected cells compared to cells infected with AAV NT4-TAT, thus confirming the expression of the CBD3 peptide.

Immunohistochemical staining with Aβ1–42 showed that, compared with control mice, APP/PS1 mice showed typical amyloid-beta aggregation and obvious senile plaques (Fig. 1b).

### CBD3 overexpression improves learning and memory abilities of APP/PS1 mice

**Fig. 2:**
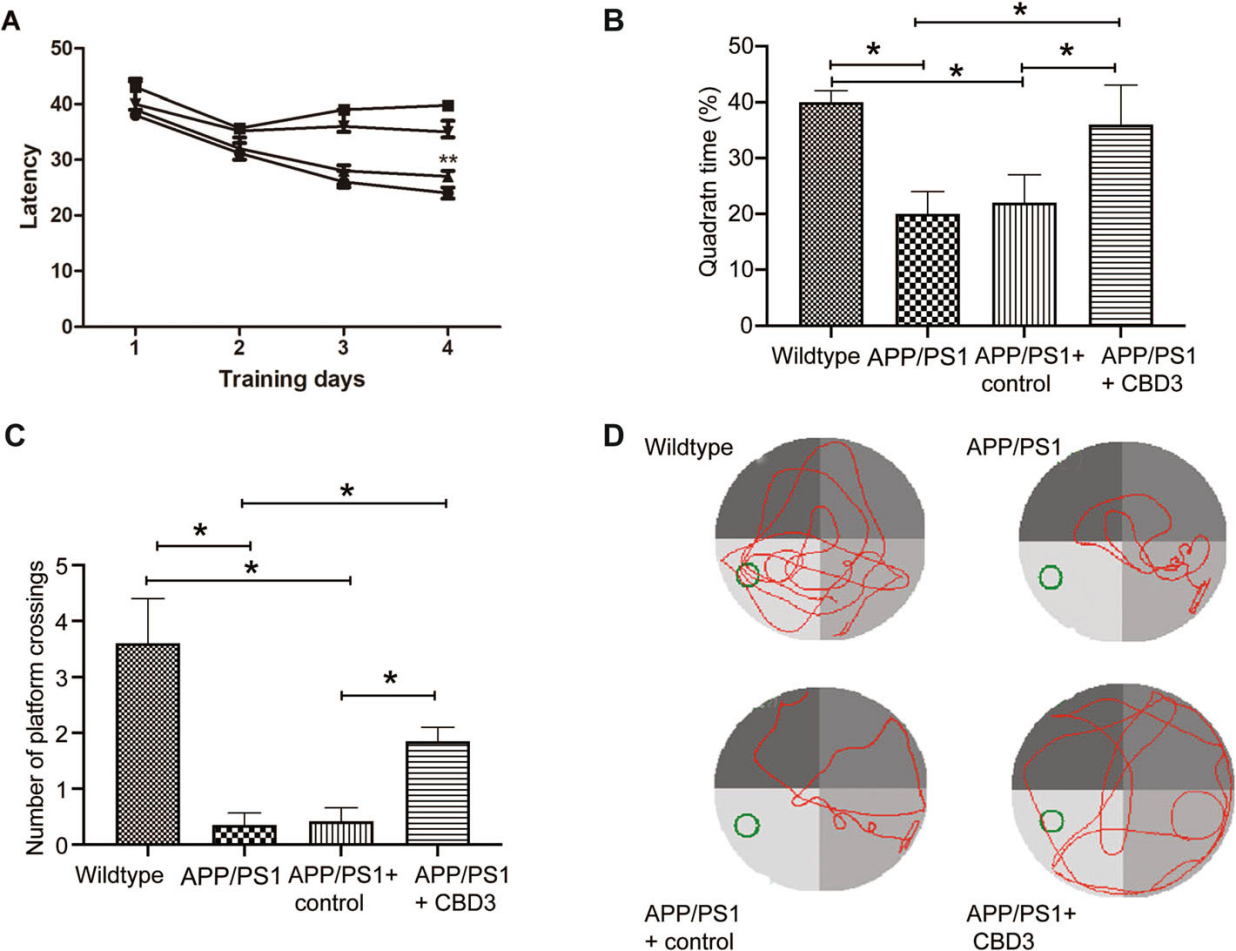
CBD3 counteracts the cognitive decline in APP/PS1 mice in Morris water maze test. **a** The escape latency (in minutes) of the WT or APP/PS1 mice treated with nothing, with AAV carrying NT4-TAT or AAV carrying NT4-TAT-CBD3 in the spatial learning test (*n* = 6). **b** The percentage of time spent in the target quadrant in the probe test (*n* = 6). **c** The number of crossings through the target quadrant (where the platform was previously located) in the probe test (*n* = 6). **d** Representative path tracings in each quadrant. The green circle represents the submerged platform. *P < 0.05, one-way ANOVA with Dunnett’s post-hoc test

In order to determine the potential therapeutic benefit of CBD3 overexpression for APP/PS1 mice cognitive function, the MWM test was conducted to investigate spatial learning and memory ability. These tests were conducted 9 days after the second nasal administration of the viral vectors (see Methods). APP/PS1 mice spent more time locating the platform when compared to wildtype (WT) mice, supporting previous findings that the cognitive ability of APP/PS1 mice in spatial learning is significantly reduced. APP/PS1 mice administered AAV overexpressing CBD3 had an escape latency from the submerged platform that was significantly better (i.e. faster) than APP/PS1 mice administered AAV lacking CBD3 (i.e. control virus). These data indicate that spatial memory was significantly improved by CBD3 overexpression (*P* < 0.05; Fig. 2a). APP/PS1 mice took a longer time to reach the missing platform and had fewer crossings into the target quadrant than control mice (Fig. 2b–d). Conversely, when compared with APP/PS1 mice or APP/PS1 mice administered AAV lacking CBD3, APP/PS1 mice administered AAV overexpressing CBD3 spent a longer time in the target quadrant and had increased crossovers into the target quadrant (Fig. 2b–d).

### CBD3 expression reduces Aβ1–42 and p-tau plaques

**Fig. 3:**
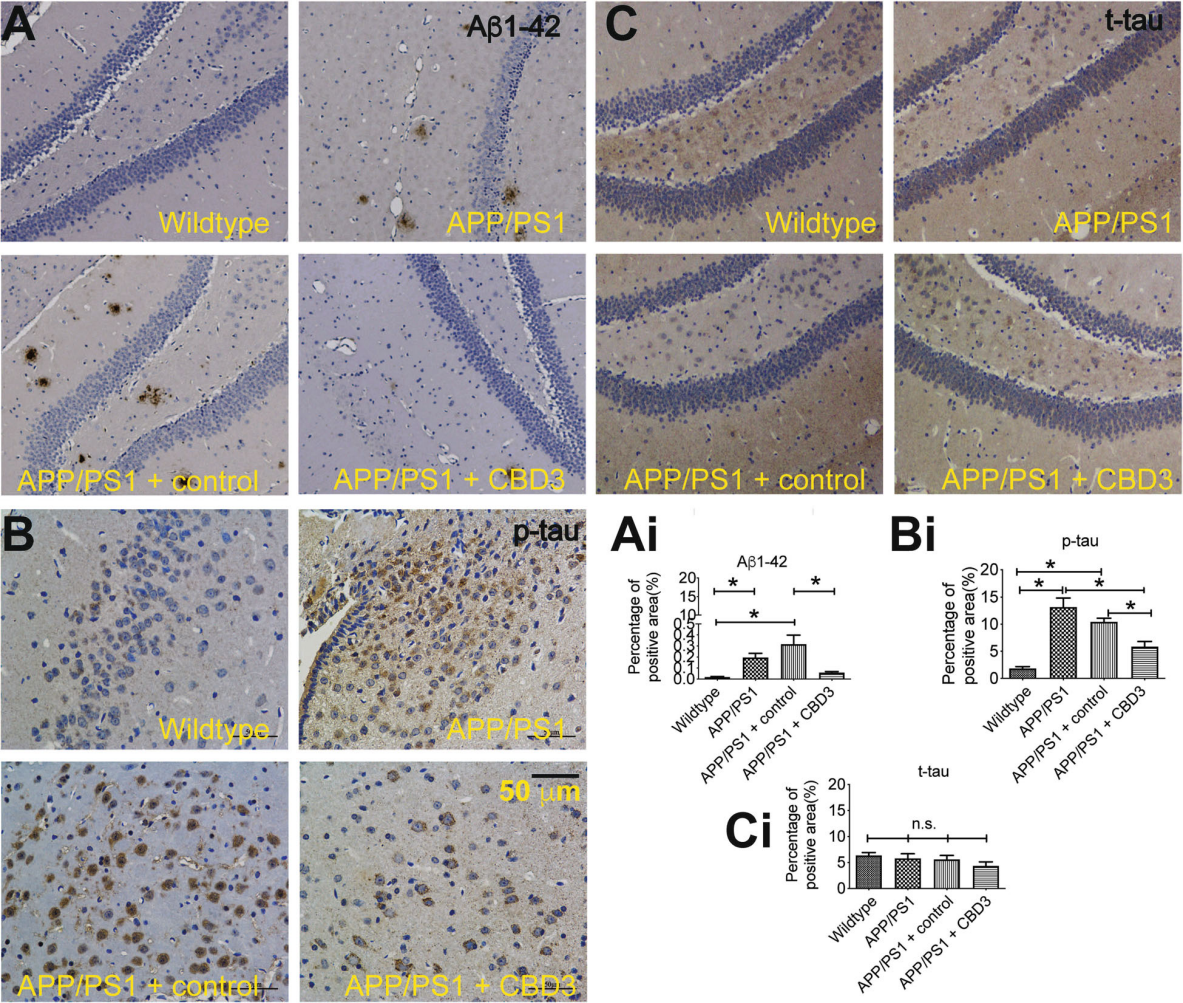
CBD3 expression reduces Aβ1–42 and p-tau plaques. Representative micrographs of hippocampal sections stained with Aβ1–42 (**a**), phosphorylated-tau (p-tau) (**b**) and total-tau (t-tau) (**c**). The hippocampal sections are from wildtype (WT) mice, APP/PS1 mice, APP/PS1 mice administered control AAV, or APP/PS1 mice administered CBD3 harboring AAV. Sections were from 3 to 4 months old mice and taken 9 days after administration of the virus nasally. Representative of *n* = 3 for age-matched control and *n* = 3 for the APP/PS1 mice. *P < 0.05, one-way ANOVA with Dunnett’s post-hoc test. n.s., not significant

To explore the effect of CBD3 on AD pathophysiology, we performed immunohistochemistry on hippocampal tissue to examine the levels of Aβ1–42, total tat (t-tau), and phosphorylated tau (p-tau) levels. Our rationale for measuring these is based on the amyloid cascade hypothesis, which purports that APP is normally cleaved by α-secretase and aberrantly processed by β- and γ-secretases resulting in an imbalance between production and clearance of Aβ peptide. As a consequence, Aβ peptides spontaneously aggregate into soluble oligomers and coalesce to form fibrils insoluble beta-sheet conformation and are eventually deposited in diffuse senile plaques. It has also been reported that the Aβ42 oligomers induce oxidative damage and promote tau hyperphosphorylation. As illustrated in Fig. 3a and Ai, the Aβ1–42 levels in APP/PS1 mice were higher than those in the WT mice. Moreover, following CBD3 overexpression in APP/PS1 mice administered AAV containing CBD3, the number of Aβ1–42-positive plaques decreased in comparison to the number of plaques found in WT mice, the APP/PS1 mice or APP/PS1 mice administered AAV lacking CBD3. Similarly, when compared with APP/PS1 mice or APP/PS1 mice administered AAV lacking CBD3, p-tau levels in APP/PS1 mice administered AAV containing CBD3 were reduced (Fig. 3b, Bi). However, there were no differences observed in t-tau levels across the four groups (Fig. 3c, Ci).

### CBD3 expression reduces hippocampal Aβ1–42 and p-tau protein levels

**Fig. 4:**
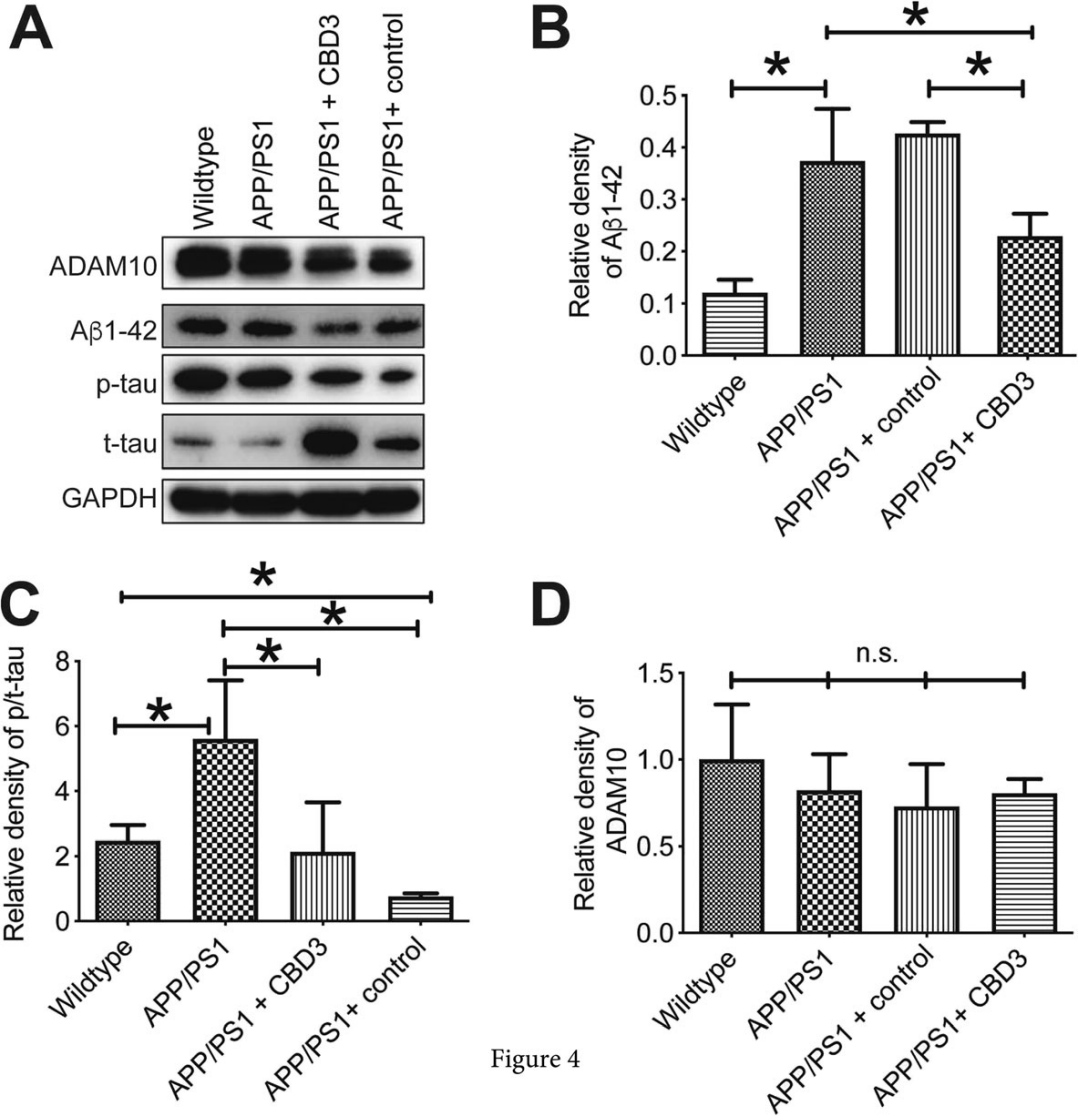
CBD3 expression reduces Aβ1–42 and p-tau plaques. Representative western blots of lysates (*n* = 4) from the indicated conditions probed with antibodies against ADAM10, Aβ1–42, p-tau, t-tau, or GAPDH (housekeeping/loading control). The hippocampal tissues were from wildtype (WT) mice, APP/PS1 mice, APP/PS1 mice administered control AAV, or APP/PS1 mice administered CBD3 harboring AAV. Quantification of levels of Aβ1–42 (**b**), p-tau/t-tau (**c**), and ADAM10 (**d**) normalized to control (*n* = 4 per condition). *P < 0.05, one-way ANOVA with Dunnett’s post-hoc test. n.s., not significant.

Western blotting was performed to quantify Aβ1–42, p-tau/t-tau, and a disintegrin and metalloproteinase 10 (ADAM10) levels in AD mice hippocampus; ADAM10 has been identified as the constitutive α-secretase in the process of amyloid-β protein precursor (AβPP) cleavage and plays a critical role in reducing the generation of the amyloid-β (Aβ) peptides. Aβ1–42 levels were higher in APP/PS1 mice compared to WT mice (Fig. 4a, b). Overexpressing CBD3 in APP/PS1 mice normalized Aβ1–42 to WT levels while APP/PS1 mice administered AAV lacking CBD3 had Aβ1–42 levels similar to those in untreated APP/PS1 mice (Fig. 4a, b). CBD3 The ratio of phosphorylated to total tau (p-tau/t-tau) protein was higher in hippocampal tissue from APP/PS1 mice compared to WT mice (Fig. 4a, c), but forcing CBD3 overexpression in APP/PS1 mice significantly reduced p-tau/t-tau levels compared with to APP/PSI mice or APP/PS1 mice administered AAV lacking CBD3 (Fig. 4a, c). These data are in agreement with the immunohistochemical results obtained earlier. ADAM10 levels, despite trends towards a decrease in APP/PS1 mice administered AAV containing CBD3, were not significantly changed across the groups (Fig. 4a, d).

### CBD3 decreased hippocampal cell apoptosis

**Fig. 5:**
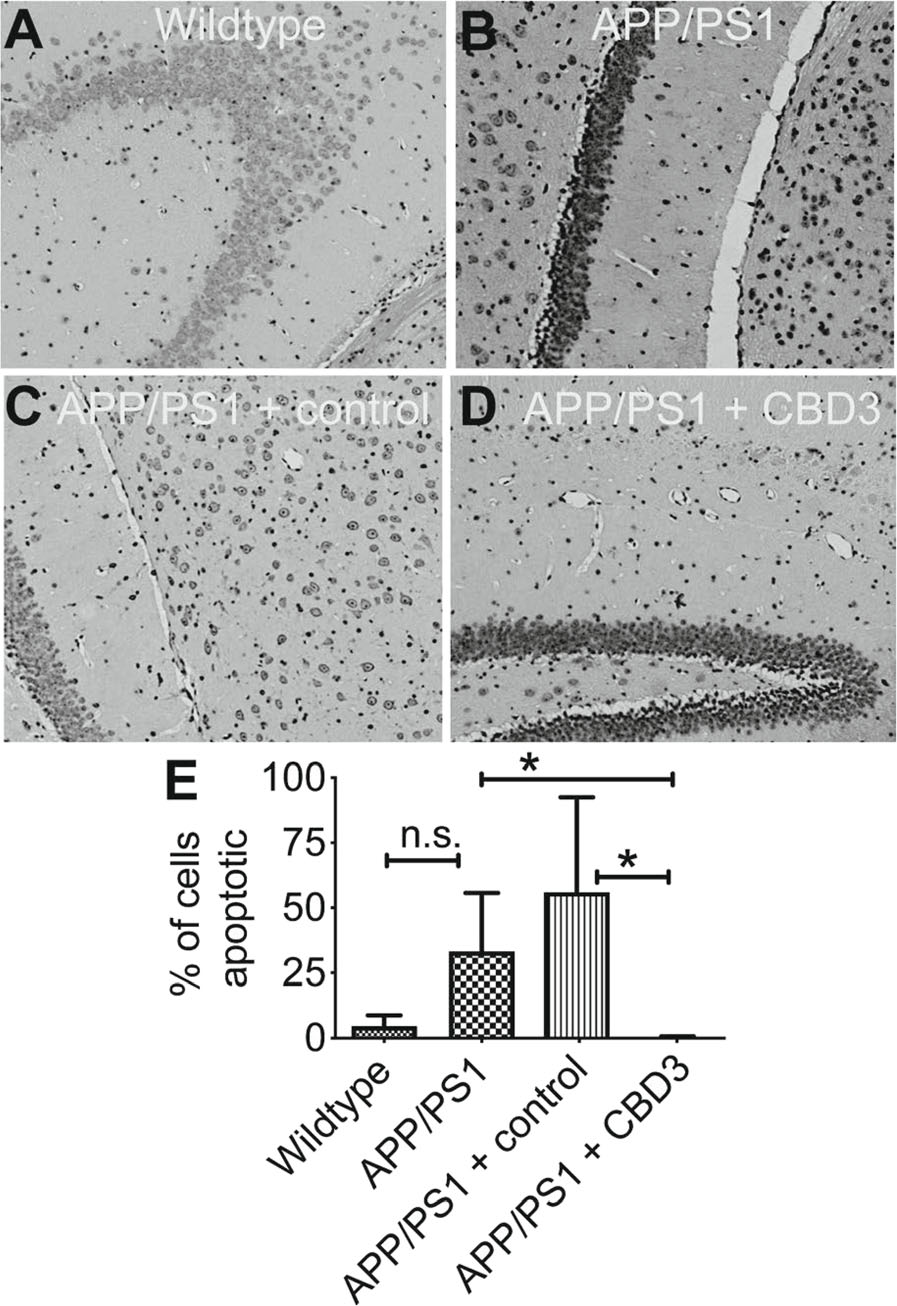
Apoptosis within the hippocampus of AD brains. **a** Representative grayscale images of TUNEL staining of CA1–2 transitional field of age-matched wildtype (WT) or APP/PS1 mice brains. The hippocampal sections are from wildtype (WT) mice, APP/PS1 mice, APP/PS1 mice administered control AAV, or APP/PS1 mice administered CBD3 harboring AAV. **b** Quantification of the percent of apoptotic cells in the indicated conditions. CBD3 overexpression reduced neuron apoptosis in APP/PS1 transgenic mice. The number of TUNEL-positive cells counted in CBD3 group was significantly lower than those in model and vector groups. Representative of *n* = 3 for age-matched control and *n* = 3 for the APP/PS1 mice. *P < 0.05, one-way ANOVA with Dunnett’s post-hoc test. n.s., not significant

Neuronal apoptosis is a pathological hallmark of AD. Aβ1–42 levels are deemed to play a central role in neuronal cell death. Since Aβ1–42 levels in APP/PS1 were normalized to wildtype by CBD3 overexpression, we asked if CBD3 was also neuroprotective. We used the TUNEL assay to detect hippocampal cell apoptosis. As shown in Fig. 5a, the brown-yellow nuclei denote TUNEL-positive cells, while the blue nuclei indicate total cells. The number of apoptotic cells was significantly higher in hippocampus of APP/PS1 mice compared to WT mice (Fig. 5b). The number of apoptotic cells was significantly lower in APP/PS1 mice administered AAV containing CBD3 compared to APP/PS1 mice or APP/PS1 mice administered AAV lacking CBD3 (Fig. 5b).

## Discussion

AD is a neurodegenerative disease characterized by cognitive decline and progressive memory deficits. Dysregulation of calcium – both via the “calcium overload hypothesis” and the “source specificity hypothesis” – have been invoked to explain the involvement of toxic levels of Ca^2+^ in AD [5, 6]. Our work on calcium-regulatory proteins led to the identification of CRMP2, an axonal specification protein that interacts with the NMDAR and regulates its function and trafficking [9, 12, 13, 58]. We further identified a 15 amino acid cytosolic fragment of CRMP2, a peptide called CBD3, which was neuroprotective against glutamate-induced excitotoxicity via actions on NMDAR currents [9]. Notably, a cell penetrant form of the peptide (TAT-CBD3): (1) induced NR2B internalization in dendritic spines without altering somal NR2B surface expression; (2) reduced NMDA-mediated Ca^2+^ influx and currents in cultured neurons; and (3) decreased hippocampal neuronal death in the controlled cortical impact model of traumatic brain injury [9]. A recent study reported that disrupting the CRMP2-NR2B association decreases memory improvement in vitamin D-treated 3xTg-AD mice [34]. Because Aβ_25–35_ oligomers can directly trigger NMDA receptor function resulting in elevated intracellular Ca^2+^, they have been associated with inflicting neuronal toxicity or death [55]. Thus, we hypothesized that by interfering with CRMP2-NMDAR interactions, we may prevent neuronal cell death and associated pathology.

That TAT-CBD3 peptide is also broadly antinociceptive in diverse models of neuropathic pain [10, 17, 40, 47] supports therapeutic targeting of CRMP2’s interactions with calcium-regulatory proteins. Of relevance to this work, an adeno-associated viral form of the CBD3 peptide was recently advanced as a potential gene therapy for chronic pain [21, 63], supporting the long-term utility of manipulating CRMP2’s interactions in neurodegenerative disease, for example. Here, we describe the potential therapeutic benefit of an AAV-form of the CBD3 peptide in amelioration of cognitive impairment, reduction of neuronal apoptosis, and normalization of the exaggerated levels of AD-related proteins, such as Aβ1–42, p-tau, and t-tau using the APP/PS1 mouse model.

Due to its increased propensity for presence of soluble and insoluble Aβ and Aβ plaque contents [32, 38], the APP/PS1 mouse is regarded as a good model for mimicking the process of AD and exploring the potential therapeutic effects of anti-AD agents [4]. The hippocampus, an area of the brain responsible for cognitive dysfunction and memory storage, is the main focus of neuron loss in AD [54]. The Morris water maze test, which relies primarily on hippocampal function, has been widely used for investigating mouse behaviors related to memory and learning [1, 30, 56]. In this study, APP/PS1 mice were used for investigating the role of CBD3 in AD progression. Immunohistochemical analysis showed that APP/PS1 model mice demonstrate typical Aβ aggregation and plaques. Moreover, the results of the Morris water maze test revealed that mice treated with CBD3 had decreased escape latency, as well as observably longer time spent in the target quadrant and increased crossovers. The data suggest that CBD3 overexpression can ameliorate the negative effects of AD pathophysiology on spatial learning and memory in APP/PS1 mice.

Many biomarkers, such as Aβ1–42, p-tau, and t-tau, are widely used to investigate different aspects of AD pathology. Briefly, amyloid plaque deposition leads to decreased concentrations of Aβ1–42; t-tau reflects neuronal degeneration; and p-tau correlates with neurofibrillary tangle formation [7, 49]. Until now, an increasing number of reports have revealed that CSF biomarkers, such as Aβ1–42, t-tau, and p-tau may be able to distinguish AD dementia patients from healthy controls [43]. To further investigate the effects of CBD3 on AD development at the molecular level, Aβ1–42, p-tau, and t-tau levels were determined in our study. Both immunohistochemical staining and western blotting showed that Aβ1–42 and p-tau/t-tau levels in APP/PS1 mice treated with AAV carrying the CBD3 cargo were observably lower than those in untreated APP/PS1 mice or APP/PS1 mice carrying AAV without CBD3. The data suggested that CBD3 could inhibit Aβ deposit and p-tau/t-tau levels in APP/PS1 mice to alleviate AD aggravation.

Furthermore, the TUNEL assay suggested that CBD3 overexpression could significantly reduce the number of apoptotic cells in AD mice hippocampus. These results are in line with previous findings demonstrating preservation of neurons in the hippocampus and granule cell layers following an intraperitoneal injection of TAT-CBD3 following a traumatic injury [9] as well as neuroprotection in an animal model of focal cerebral ischemia [11]. Varying the cell-penetrating motif from TAT to a non-arginine (R9)-conjugated CBD3 peptide similarly inhibited cellular apoptosis in Aβ-induced injury [28]. However, studies on the effect of CBD3 on cellular apoptosis in AD are limited. Our data revealed that CBD3 may inhibit AD development by regulating cellular apoptosis.

In conclusion, our data in the APP/PS1 mouse model demonstrated that CBD3 could promote spatial learning and memory in AD mice. Furthermore, CBD3 overexpression inhibits AD development by decreasing Aβ deposit and p-tau/t-tau levels, as well as decreasing hippocampal cell apoptosis. Our findings provide evidence for a protective effect of CBD3 against AD, implicating the CRMP2 protein as a potential therapeutic target for AD. In support of this assertion, genetically interfering with CRMP2 phosphorylation on S522 prevented Aβ-mediated impairment of long-term potentiation (LTP) [27]. A number of Cdk5, GSK3β, and CRMP2 phosphorylation blocking compounds have been used with varying success in AD models, resulting in fewer and smaller aggregates, improved memory and learning, and improved synaptic signaling [27, 59–61].

In AD, recent evidence suggests that the spread of the disease relies on the trans-synaptic transfer of pathological Tau and other prion-like proteins [42]. This raises the hypothesis that trans-synaptic transfer of (phosphorylated) CRMP2 in AD could be participating in this prion-like propagation of the pathology. This is based on the recent findings of an extracellular pool of CRMP2, which acts as an agonist for NMDA receptors [15, 39]. However, further studies will be needed to understand the mechanisms and consequences of extracellularly localized CRMP2.

## Supplementary information

**Additional file 1.**

## Data Availability

Please contact author for data requests.
