# Supplementary figures and images for: Nasal delivery of a CRMP2-derived CBD3 adenovirus improves cognitive function and pathology in APP/PS1 transgenic mice
Source: Mol Brain. 2020 Apr 9;13:58. doi: 10.1186/s13041-020-00596-3 (PMC7144060; doi:10.1186/s13041-020-00596-3)

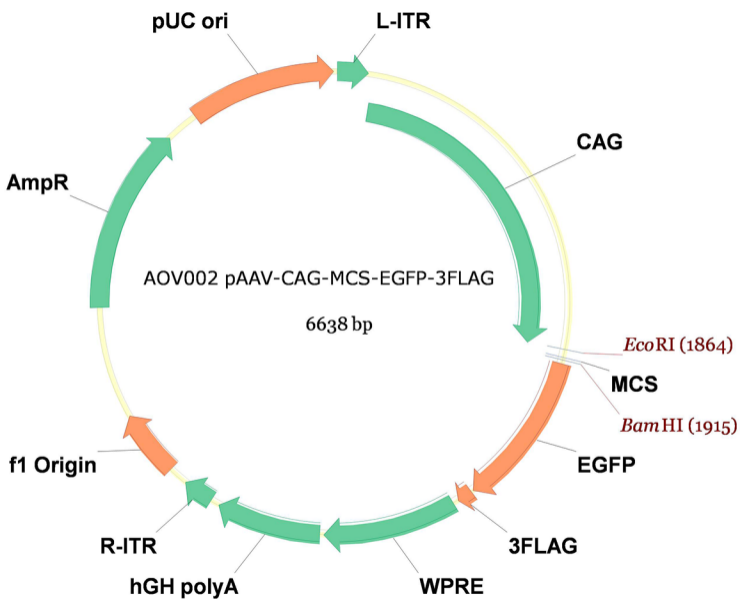

Supplement: Supplementary file 1 — Additional file 1. [file 13041_2020_596_MOESM1_ESM.pdf]
